# Rice bacterial blight pathogen *Xanthomonas oryzae *pv. *oryzae *produces multiple DSF-family signals in regulation of virulence factor production

**DOI:** 10.1186/1471-2180-10-187

**Published:** 2010-07-09

**Authors:** Ya-Wen He, Ji'en Wu, Jae-Soon Cha, Lian-Hui Zhang

**Affiliations:** 1 Institute of Molecular and Cell Biology, 61 Biopolis Drive, 138673, Singapore; 2 Department of Plant Medicine, Chungbuk National University, Cheongju 361-763, Korea

## Abstract

**Background:**

*Xanthomonas oryzae *pv. *oryzae *(*Xoo*) is the causal agent of rice bacterial blight disease. *Xoo *produces a range of virulence factors, including EPS, extracellular enzyme, iron-chelating siderophores, and type III-secretion dependent effectors, which are collectively essential for virulence. Genetic and genomics evidence suggest that *Xoo *might use the diffusible signal factor (DSF) type quorum sensing (QS) system to regulate the virulence factor production. However, little is known about the chemical structure of the DSF-like signal(s) produced by *Xoo *and the factors influencing the signal production.

**Results:**

*Xoo *genome harbours an *rpf *cluster comprising *rpfB*, *rpfF*, *rpfC *and *rpfG*. The proteins encoded by these genes are highly homologous to their counterparts in *X. campestris *pv. *campestris *(*Xcc*), suggesting that *Xcc *and *Xoo *might use similar mechanisms for DSF biosynthesis and autoregulation. Consistent with *in silico *analysis, the *rpfF *mutant was DSF-deficient and the *rpfC *mutant produced about 25 times higher DSF-like activity than the wild type *Xoo *strain KACC10331. From the supernatants of *rpfC *mutant, we purified three compounds showing strong DSF-like activity. Mass spectrometry and NMR analysis revealed that two of them were the previously characterized DSF and BDSF; the third one was a novel unsaturated fatty acid with 2 double bonds and was designated as CDSF in this study. Further analysis showed that all the three DSF-family signals were synthesized via the enzyme RpfF encoded by *Xoo2868*. DSF and BDSF at a final concentration of 3 μM to the *rpfF *mutant could fully restore its extracellular xylanase activity and EPS production to the wild type level, but CDSF was less active than DSF and BDSF in induction of EPS and xylanase. DSF and CDSF shared a similar cell density-dependent production time course with the maximum production being detected at 42 h after inoculation, whereas the maximum production of BDSF was observed at 36 h after inoculation. When grown in a rich medium such as YEB, LB, PSA, and NYG, *Xoo *produced all the three signals with the majority being DSF. Whereas in nutritionally poor XOLN medium *Xoo *only produced BDSF and DSF but the majority was BDSF.

**Conclusions:**

This study demonstrates that *Xoo *and *Xcc *share the conserved mechanisms for DSF biosynthesis and autoregulation. *Xoo *produces DSF, BDSF and CDSF signals in rich media and CDSF is a novel signal in DSF-family with two double bonds. All the three DSF-family signals promote EPS production and xylanase activity in *Xoo*, but CDSF is less active than its analogues DSF and BDSF. The composition and ratio of the three DSF-family signals produced by *Xoo *are influenced by the composition of culture media.

## Background

The quorum sensing (QS) mechanism allows bacteria to sense their population density and synchronize individual activity into cooperative community behaviour [1-3], which appears to provide bacterial pathogens an obvious competitive advantage over their hosts in pathogen-host interaction. In Gram-negative bacteria, in addition to the well-characterized AHL-type QS signals and AI-2, DSF-family signals have recently been reported in a range of plant and human bacterial pathogens, including *Xanthomonas campestris *pv. *campestris *(*Xcc*), *Xyllela fastidiosa*, *Stenotrophomonas maltophilia*, and *Burkholderia cenocepacia *[4-9]. In *Xcc*, DSF has been characterized as cis-11-methyl-2-dodecenoic acid [5]. The putative enoyl-CoA hydratase RpfF is a key enzyme for DSF biosynthesis [4,10]. The DSF signalling system comprises several key regulatory proteins and a second messenger cyclic-di-GMP (c-di-GMP). Among them, the RpfC/RpfG two-component system is involved in sensing and transduction of DSF signal through a conserved phosphorelay mechanism [10-12]; RpfG functions in turnover of the second messenger c-di-GMP and Clp is a novel c-di-GMP receptor [12,13], which regulates the expression of DSF-dependent genes directly or indirectly via two downstream transcription factors Zur and FhrR [14]. In *Xylella fastinosa*, the structure of the DSF-like signal was characterized tentatively as 12-methyl-tetradecanoic acid by high-resolution gas chromatography-mass spectrometry (HRGC-EI-MS) analysis [6]. The DSF-like signal molecule (BDSF) from *B. cenocepacia *has been purified and characterized as *cis*-dodecenoic acid [9]. Moreover, DSF and several extracellular fatty acids have been identified from *S. maltophilia *by electrospray ionization mass spectrometry (ESI-MS) and gas chromatography and mass spectrometry (GC-MS analysis) [7]. Functional analysis of *rpfF *or *rpfC *mutants in different bacterial species suggests that the general role of the DSF-signaling system in the modulation of virulence seems to be conserved, but the regulatory mechanisms and DSF-dependent traits may differ among taxa [8,15-17].

*Xanthomonas oryzae *pv. *oryzae *(*Xoo*) is a causal agent of bacterial blight disease of rice [18]. *Xoo *enters either through wounds or hydathodes, multiplies in the epitheme and moves to the xylem vessels where active multiplication results in blight disease symptoms on rice leaves. Similar to *Xcc*, *Xoo *also produces a range of virulence factors, including EPS, extracellular enzyme, iron-chelating siderophores, and the type III-secretion dependent effectors, which are collectively essential for virulence [19-23]. Null mutation of *rpfC *in *Xoo *wild type strain T3000 substantially affects the EPS synthesis and virulence [24]. The *rpfF *mutants of an Indian *Xoo *wild type isolate BXO43 are attenuated in virulence and defective in growth under low iron conditions [15]. More recently, a report showed that mutations in the core *rpf *genes *rpfB*, *rpfF*, *rpfC *and *rpfG *reduced the EPS levels, xylanase activity, motility, and virulence of *Xoo *strain KACC10331 [25]. These findings suggest that DSF signalling system in *Xoo *is involved in the regulation of virulence factor production. However, little is known about the chemical structure of the DSF-family signals in *Xoo *and the factors influencing the signal production.

In this study, the comparative genomics analysis revealed that *Xoo *genome shares the key components of DSF biosynthesis and signalling with *Xcc*. The DSF production assay of *rpfF*, *rpfC*, *rpfG *mutants showed that *Xoo *uses a similar autoregulation mechanism as *Xcc *to control DSF biosynthesis. We further found that *Xoo *produces three DSF-family signals: DSF, BDSF and a novel signal with two double bonds, which was designated as CDSF. All the three DSF-family signals induce the EPS production and extracellular xylanase activity in the *rpfF *mutant of *Xoo *with variable efficiencies. Moreover, we found that the production and the ratio of the DSF-family signals are affected by the culture medium composition.

## Results

### *Xoo *uses the similar mechanism of *Xcc *in autoregulation of DSF biosynthesis

**Figure 1:**
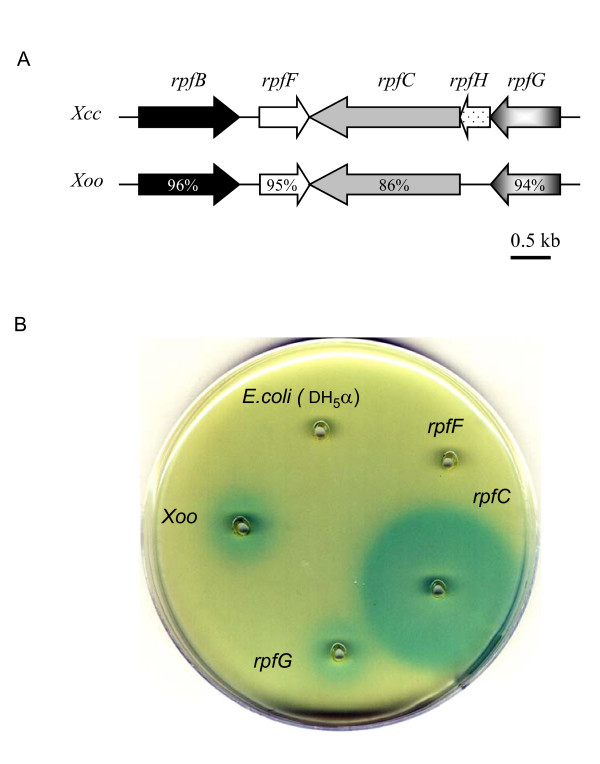
***Xoo *and *Xcc *share conserved mechanisms for DSF biosynthesis autoregulation**. (A) Physical map of the part of the *rpf *gene cluster from *rpfB *to *rpfG *in *Xoo *strain KACC10331 and *Xcc *strain ATCC33913. The organization of ORFs predicted by sequence analysis together with predicted directions of transcription are indicated by the broad arrows. (B) DSF production of *Xoo *strain KACC10331 and derivatives.

In *Xcc*, the *rpf *cluster is involved in DSF biosynthesis, signal sensing and response. RpfF, a putative enoyl-CoA hydratase, is a key enzyme involved in DSF biosynthesis and mutation of *rpfF *abolishes DSF production [4]. RpfC negatively controls DSF biosynthesis by binding to RpfF at low cell density [10], and disruption of *rpfC *results in a 16-fold higher DSF accumulation than the wild-type *Xcc *[5,11]. The genomes of three sequenced *Xoo *strains (KACC10331, MAFF311018 and PX099A) contain the *rpf *cluster comprising *rpfB*, *rpfF*, *rpfG *and *rpfC*, but not *rpfH *[26-28]. These *rpf *homologous from *Xcc *and *Xoo *share more than 86% identify at the amino acids level (Fig. 1A), suggesting the conserved mechanism in DSF biosynthesis and in DSF signalling. To confirm this possibility, the *rpfF*, *rpfC *and *rpfG *mutants of *Xoo *strain KACC 10331, which were described previously [25], were assayed for DSF production. The results showed that the *rpfF *mutant is DSF-deficient while the *rpfC *mutant produced DSF signal around 25 times higher than its wild type parental strain did (Fig. 1B). The DSF production patterns of *rpfC*, *rpfF *and *rpfG *mutants of *Xoo *were very similar to those of *Xcc *[5,10,11], which indicates that, similar to XC1, *Xoo *also uses the RpfC-RpfF protein-protein interaction mechanism to autoregulate the biosynthesis of DSF-like signals.

### *Xoo *produces multiple DSF-family signals

**Figure 2:**
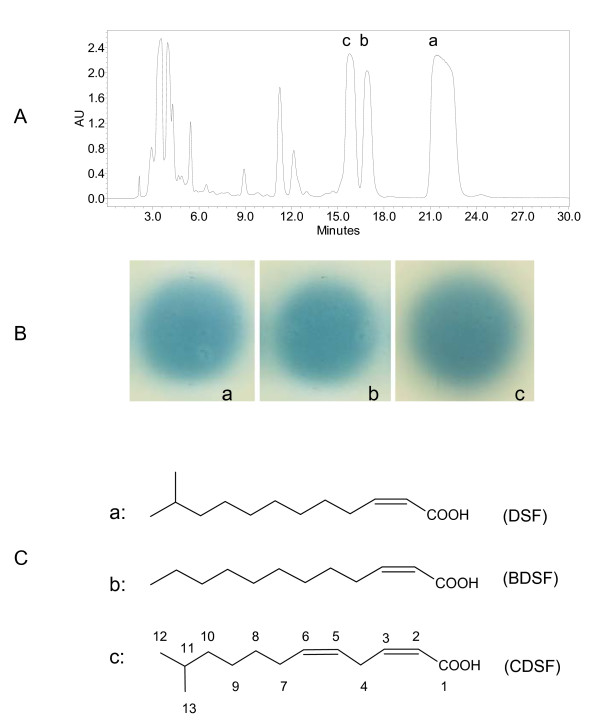
***Xoo *produces multiple DSF-family signals**. (A) HPLC analysis of the active fractions after flash column chromatography. (B) The compounds in fractions a, b, and c showed strong DSF-like activity. (C) Chemical structures of the compounds in fractions a, b, and c as confirmed by ESI-MS and NMR analysis.

To identify the DSF-like signals produced by *Xoo*, we prepared the DSF extracts from the culture supernatants of the *rpfC *mutant using a similar method as previously described [5] with two minor modifications. Firstly, we adjusted the pH of the supernatants of *Xoo *cell culture to 4.0 using concentrated hydrochloric acid before extraction by ethyl acetate. Secondly, formic acid was added at a final concentration of 0.1% to all the solvents for purification and high-performance liquid chromatography (HPLC) analysis. By using the DSF bioassay system described by Wang *et al*. [5], active fractions were collected and combined following flash column chromatography. Further separation using HPLC identified three active fractions with retention time at 15.7, 17.0, and 21.4 min, respectively, showing a maximum UV absorption at 212 nm and strong DSF activity in bioassay (Fig. 2A-B). High-resolution electrospray ionization mass spectrometry (ESI-MS) and NMR analysis showed that the compound in fraction A was *cis*-11-methyl-2-dodecenoic acid (DSF) (Additional file 1), which was originally reported in *Xcc *by Wang *et al*. [5]. The compound in fraction B showed the same NMR spectra and molecular weight as the BDSF signal from *Burkholderia cenocepacia *[9] (Additional file 2). The spectrometry data of fraction C suggested a new member of the DSF-family signals (designated as CDSF) and its characterization was discussed in the following section.

### CDSF is a novel DSF-family signal

**Figure 3:**
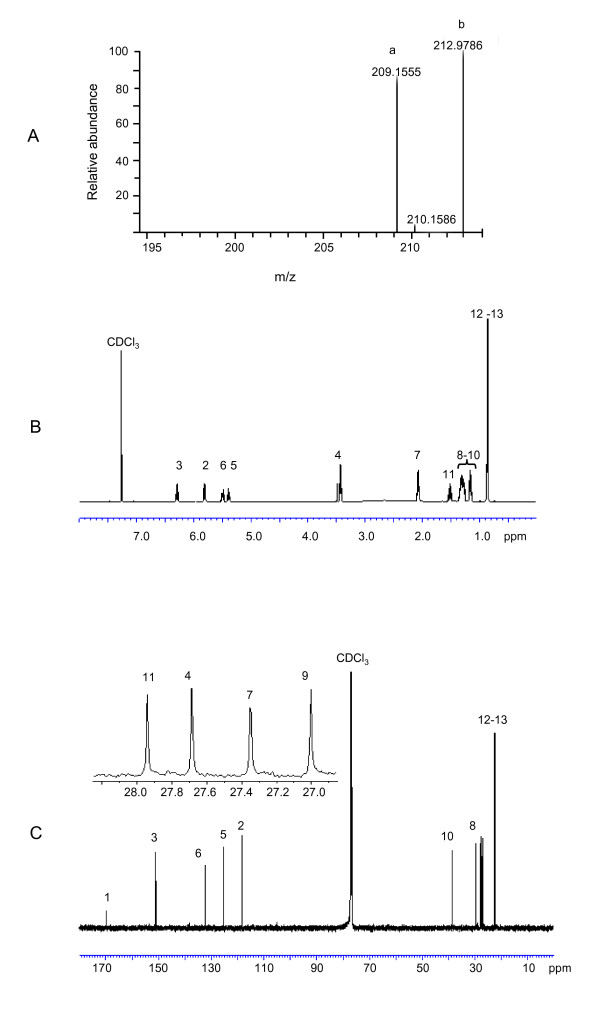
**CDSF is a novel DSF-family signal**. (A) High resolution ESI-MS analysis of CDSF showing a molecular weight of 209.1555 dalton (peak a). The internal control was indicated as peak b. (B) The ^1 ^H NMR spectra of CDSF. (C) The ^13 ^C NMR spectra of CDSF. The NMR analyses were conducted at room temperature (CDCl_3_, 125MHz).

High resolution MS analysis of CDSF showed a moleclar ion (M-H)^- ^with a *m/z *of 209.1555, suggesting a molecular formula of C_13_H_21_O_2 _(209.1547) (Fig. 3A). ^1^H NMR analysis revealed two pairs of methylenic protons. The coupling constants between the protons in each pair were lower than 12 Hz (Fig. 3B), suggesting the presence of two double bonds in *cis *configuration. The δ_H _of two methylene protons were at 3.45, revealing a methylene carbon associated with two double bonds. The δ_H _of overlapped signals of two doublet methyl group were at 0.87, indicating a DSF-like branched structure. ^13^C NMR spectra analysis revealed that one double bond conjugated with the carbolic acid (Fig. 3C). Taken together, these data establish that CDSF is a novel unsaturated fatty acid, which is otherwise identical to DSF except the double bond between C_5 _and C_6 _(Fig. 2C).

### DSF, BDSF and CDSF are synthesized via RpfF in *Xoo*

Previous study showed that the signal DSF is synthesized via RpfF in *Xcc *[4]. Our results in Fig. 1B showed that deletion of *rpfF *in *Xoo *resulted in loss of DSF-like activity, suggesting that DSF, BDSF and CDSF are all synthesized by RpfF in *Xoo*. For further verification, we compared the HPLC profiles of organic solvent extracts from *Xoo *wild type and its *rpfF *mutant. The results showed that the three fractions corresponding to DSF, BDSF and CDSF were detectable from the extracts of the *Xoo *wild type but not from the *rpfF *mutant (Additional file 3).

### CDSF is a functional signal on induction of EPS production and extracellular xylanase activity

**Figure 4:**
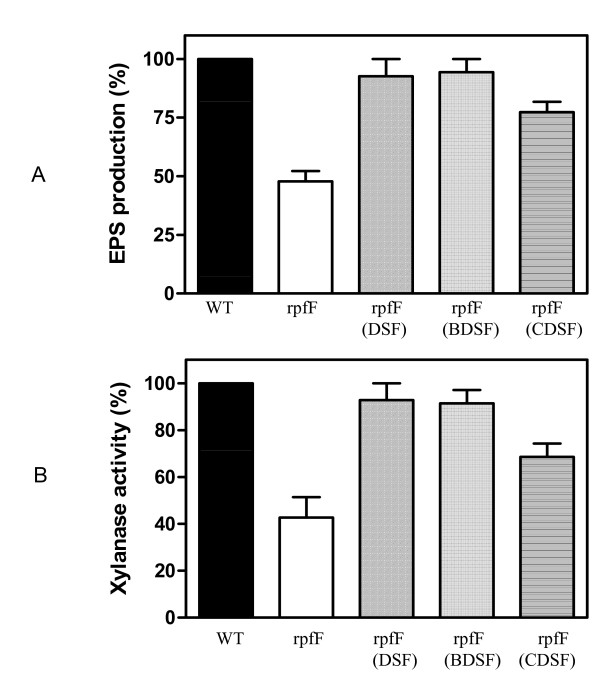
**Effects of DSF, BDSF and CDSF on EPS production and extracellular xylanase activity of *rpfF *mutant of *Xoo *strain KACC10331**. (A) EPS production at OD_600 _= 2.5. (B) The xylanase activity in the supernatant of cell culture at OD_600 _= 2.5. DSF, BDSF and CDSF were separately added to *rpfF *mutant at early growth stage at a final concentration of 3 μM.

Previous findings in *Xoo *strain KACC10331 showed that mutation in *rpfF *reduced the EPS production, xylanase activity, motility and virulence [25], suggesting the involvement of the DSF family signals in modulation of virulence factor production. In this study, the purified DSF, BDSF and CDSF were added separately to the *rpfF *mutant in a concentration range of 1 to 25 μM. After growth for 48 h, the EPS production and the extracellular xylanase activity in the supernatants were determined. The results showed that 1 μM of DSF or BDSF significantly stimulated EPS production and xylanase activity whereas 1 μM of CDSF had no effect (Additional file 4). EPS production and extracellular xylanase activity of *rpfF *mutant could be restored to wild-type level by addition of DSF or BDSF at a final concentration of 3 μM (Additional file 4; Fig. 4). CDSF at the same concentration could only restore EPS production and xylanase activity to 77.0% and 68.5% of the wild type level, respectively (Fig.4).

### Three signals were differentially produced in *Xoo*

**Figure 5:**
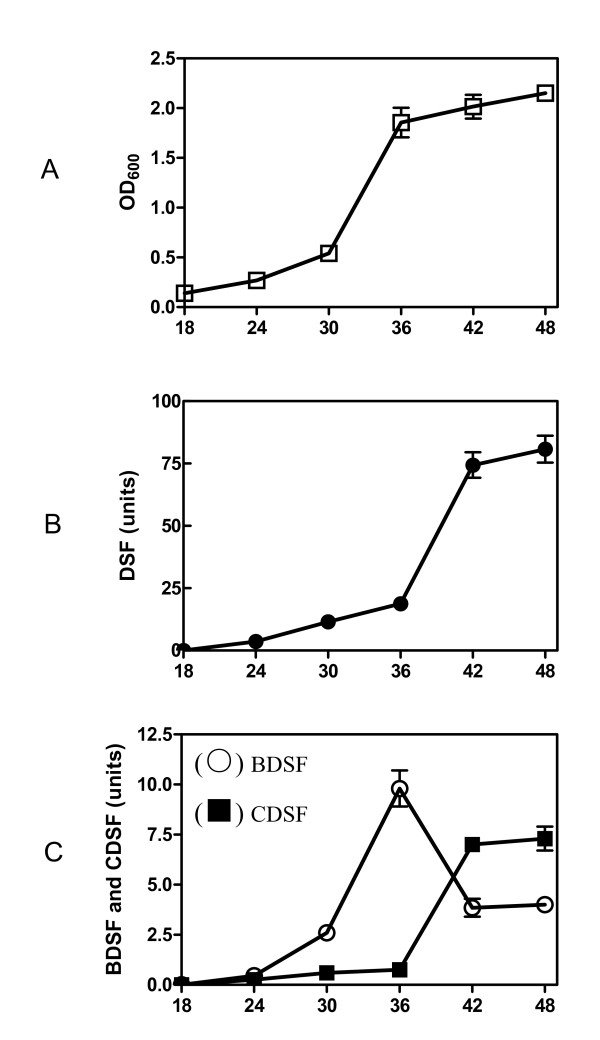
**Time course of DSF, BDSF and CDSF production in *Xoo *during growth**. (A) Time course of the bacterial growth in YEB medium. (B) Time course of DSF production. (C) Time course of BDSF and CDSF production. Units of DSF, BDSF and CDSF were determined by peak area in HPLC elute as indicated in Materials and Methods.

The maximal DSF production in *Xcc *was found to be at the late stationary phase using a bioassay approach [5]. In this study, a more sensitive HPLC method was used to determine the production profiles of the DSF-family signals in *Xoo*. The bacterial strain was grown in the same medium for 48 h as described for *Xcc *[5], and the bacterial cell density and the levels of DSF, BDSF, and CDSF in the supernatants were monitored every 6 hours. The results showed that *Xoo *strains grew relatively slow during the first 30 h and then multiplied exponentially at about 36 h after inoculation (Fig. 5A). In agreement with this trend, the DSF level remained relatively low before 36 h after inoculation and a substantial increase was observed at 42 h after inoculation (Fig. 5B). The CDSF shared a similar production pattern as DSF except that the CDSF level in the supernatants was around 10 times lower than that of DSF at 42 h after inoculation (Fig. 5C). In contrast, the BDSF level in the supernatants increased stably from 18 h after inoculation and the maximal BDSF production occurred at 36 h after inoculation (Fig. 5C). A substantial decrease in BDSF production was observed 42 h after inoculation (Fig. 5C). At 36 h after inoculation, the BDSF level in the supernatants was around 2 times lower than that of DSF (Fig. 5C).

### Influence of culture media on signal production

**Figure 6:**
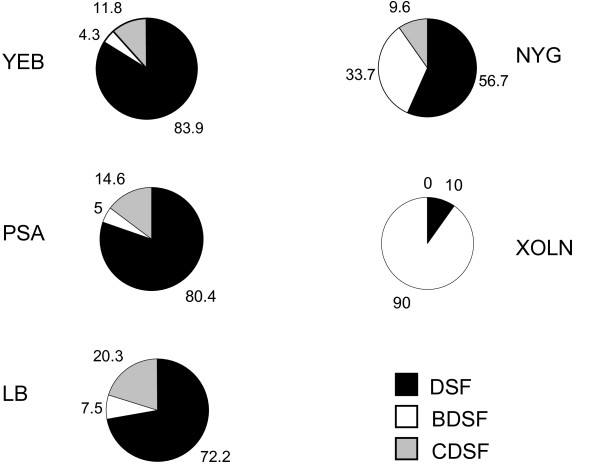
**Three signals were differentially produced in *Xoo ***. *rpfC *mutant of *Xoo *strain was grown in YEB medium for 48 h and DSF-family signals were extracted and purified from the supernatants for HPLC analysis. The relative percentage of DSF, BDSF and CDSF in one sample was determined by the percentage of peak area in HPLC elute.

The differential signal production patterns shown in Fig. 5 suggest that substrate availability may be a factor in shaping the corresponding signal production profile. As the substrate availability could be influenced by nutritional composition and growth stages, we tested whether the signal production could be affected by culture media. To this end, the *rpfC *mutant of *Xoo *strain was grown in 5 different culture media for 48 h to analyse the production of the 3 DSF-family signals. The results showed that the maximum cell density varied in different growth media. Among the 5 media tested, YEB medium supported the best bacterial growth (OD_600 _= 2.5 ± 0.2), followed by LB (OD_600 _= 2.1 ± 0.1), PSA (OD_600 _= 2.1 ± 0.1), NYG (OD_600 _= 1.9 ± 0.1) and XOLN (OD_600 _= 1.8 ± 0.1). When grown in rich media such as YEB, LB, PSA, and NYG, *Xoo *strain produced all the 3 signals with the majority being DSF ranging from 56.7 ~ 83.9% (Fig. 6). However, when cultured in the nutritionally poor XOLN medium, *Xoo *produced only DSF and BDSF with 90% of them being BDSF (Fig. 6).

## Discussion

In this study, based on the finding that DSF is a long chain fatty acid, we modified our previously developed method for DSF extraction and purification by adjusting the cell culture supernatant's pH from 7 to 4 prior to ethyl acetate extraction. The results showed that *Xoo *strain KACC10331 produces 3 DSF-family signals, including the previously characterized DSF in *Xcc *[5], BDSF in *Bcc *[9] and a novel DSF-family signal CDSF (Fig. 2). In contrast, only DSF was identified from the same volume of unacidified supernatants and its yield was about 10-fold lower than that from the acidified supernatants (data not shown). The findings encouraged us to check whether *Xcc *could also produce other DSF-family signals in addition to DSF. By using this modified protocol, we confirmed that *Xcc *also produced the same 3 signals as *Xoo *(data not shown). Taken together, these results suggest that both *Xoo *and *Xcc *produce multiple DSF-family signals, which is consistent with the previous finding that *S. maltophilia *strain WR-C produces a range of extracellular fatty acids, including DSF and seven structural derivatives [7]. It remains to be determined why and how bacteria produce multiple DSF-family signals. Although our results showed that DSF, BDSF and CDSF are all functional signals on the induction of EPS production and xylanase activity, we still could not rule out the possibility that these structurally distinct molecules might have different roles. Alternatively, these DSF-family signals might be functionally interchangeable and the mixture of them might simply be a mater of circumstance from a relatively promiscuous RpfF enzyme. The latter was further supported by the experimental findings that culture media influenced the production of DSF-family signals (Fig. 6). *Xoo *is a vascular pathogen, and the nutrients available in the xylem are probably different from those of the media used in this study. Thus, to determine what the true signal is used for *in vivo *quorum sensing during multiplication inside the vascular system of rice will be one of the key subjects of future work.

So far, little is known about the DSF biosynthesis pathway except that RpfF is the key enzyme involved in DSF biosynthesis. RpfF is predicted to be a putative enoyl-CoA hydratase, but the precursors of DSF-family signals and the mechanism of catalysis remain to be determined [29]. Given that CDSF differs from DSF in only one double bond, it is highly likely that they were not derived from one single precursor, whereas BDSF was produced from another precursor. The data from this study showed that RpfF is essential for production of DSF, BDSF and CDSF, suggesting that RpfF could accommodate and use at least two types of precursors for the synthesis of different DSF-family signals. The findings that the maximum production of BDSF occurred ahead from the other two signals suggest that these precursors are produced differentially during bacterial growth. The notion is agreeable with the observations that the medium composition affected the ratio of the 3 DSF-family signals (Fig. 6).

The previous work on *Xcc *revealed that the unsaturated double bond at the α,β position of DSF is important for it signalling activity and the saturated derivative is about 20,000 times less active than DSF [5]. BDSF is structurally different from DSF in the methyl substitution at the C-11 position (Fig. 2C). Similarly, DSF and BDSF had comparable effects on EPS production and on extracellular xylanase activity in *Xoo*, but CDSF was less active than its two analogues (Fig. 3). Presumably, the extra double bond at the C_5_-C_6 _of CDSF may affect its configuration, which hinders its accessibility to across the outer membrane or interaction with the sensor kinase. Consistent with this notion, farnesoic acid (3,7,11-trimethyl-2,6,10-dodecatrienoate), which contains two more double bonds in addition to the α,β double bond, shows a lower biological activity than DSF in *Xcc *[5]. Taken together, our results suggest that the DSF signalling mechanisms, especially at the level of the signal production autoregulation, are likely highly conserved in *Xcc *and *Xoo*.

## Conclusions

*Xoo *strain KACC10331 produces multiple DSF-family signals, including DSF, BDSF and CDSF, when grown in rich media. *Xoo *uses a similar mechanism as previously described in *Xcc *to autoregulate the biosynthesis of the DSF-family signals. All the three DSF-family molecules are active signals in induction of the virulence factor production in *Xoo *although the efficiency may vary. The amount and ratio of the DSF-family signals produced by *Xoo *are influenced by culture medium composition.

## Methods

### Bacterial strains and growth conditions

*Xoo *wild type strain KACC10331 and the derivates were described previously [25]. *Xoo *strains were routinely grown at 30°C in YEB medium with 10 μg/ml cephalexin unless otherwise stated, which comprises 5 g/L yeast extract, 10 g/L tryptone, 5 g/L sodium chloride, 5 g/L sucrose, 0.5 g/L MgSO_4_. The NYG medium comprises 5 g/L peptone, 3 g/L yeast extract and 20 g/L glycerol. PSA medium contains 10 g/L peptone, 10 g/L sucrose, and 1.0 g/L Na-glutamate. The composition of XOLN medium: K_2_HPO_4 _0.7 g/L, KH_2_PO_4 _0.2 g/L, (NH_4_)_2_SO_4 _1 g/L, MgCl_2 _0.1 g/L, FeSO_4 _0.01 g/L, MnCl_2 _0.001 g/L, 0.0625% tryptone, 0.0625% yeast extract, sucrose 2 g/L [30]. All tryptone, peptone and yeast extract were from Becton, Dickinson and Company (USA).

### Bioassay and quantification analysis of DSF-like signals

DSF bioassay and quantification was performed as described previously [5]. Briefly, *Xoo *strains were grown for 2 days until an OD_600 _of 2.3 and 25 μl of cell cultures were added to each well. The bioassay plates were incubated at 28°C for 24 hr. DSF activity was indicated by the presence of a blue halo around the well. To quantify DSF production, blue halo zone widths in the bioassay were converted to DSF units using the formula: DSF(unit ml^-1^) = 0.134 e^(1.9919W)^, where W is the width in centimeters of the blue halo zone surrounding each well. Relative level of DSF-family signals in one sample was quantified using peak area in HPLC elute. One unit of DSF was defined as 100,000 μV/sec.

### Purification of DSF, BDSF and CDSF

*Xoo *strain was cultured in YEB medium for 48 h. Five liters of bacterial supernatant were collected by centrifugation at 3,800 rpm for 30 min at 4°C (J6-HC Centrifuge, BECKMAN COULTER™). The pH of the supernatants was adjusted to 4.0 by adding hydrochloric acid prior to extraction with an equal volume of ethyl acetate twice. The ethyl acetate fractions were collected and the solvent was removed by rotary evaporation at 40°C to dryness. The residue was dissolved in 20 ml of methanol. The crude extract, divided into four batches, was subjected to flash column chromatography using a silica gel column (12 × 150 mm, Biotage Flash 12 M cartridge), eluted with ethyl acetate-hexane (25:75, v/v, 0.05% acetic acid). The collected active component was then applied to HPLC on a C18 reverse-phase column (4.6 × 250 mm, Phenomenex Luna), eluted with water in methanol (20:80, v/v, 0.1% formic acid) at a flow rate of 1 ml/min in a Waters 2695 system with 996 PDA detector.

### Structure analysis

^1^H, ^13^C, ^1^H-^1^H COSY, and heteronuclear multiple quantum coherence (HMQC) nuclear magnetic resonance (NMR) spectra in CDCl_3 _solution were obtained using a Bruker DRX500 spectrometer operating at 500 MHz for ^1^H or 125 MHz for ^13^C. High-resolution electrospray ionization mass spectrometry was performed on a Finnigan/MAT MAT 95XL-T mass spectrometer.

### Quantitative determination of extracellular xylanase activity and EPS production

The fresh colonies of *Xoo *strains were inoculated in 50 ml of YEB liquid medium with or without DSF-family signals at a starting OD_600 _of 0.05. After growth for two days, the bacterial cultures at an OD_600 _of 2.5 were collected and the supernatants were prepared by centrifugation at 14,000 rpm for 10 min. The extracellular xylanase activity in the culture supernatants of *Xoo *strains were measured by using 4-O-methyl-D-glucurono-D-xylan-Remazol Brilliant Blue R (RBB-Xylan; Sigma Co.) according to the methods described previously [31,25]. To determine the production of EPS, potassium chloride was added to 10 ml of the supernatants at a final concentration of 1.0% (w/v). Two volumes of absolute ethanol were added to the supernatants and the mixtures were then kept at -20°C for overnight. The precipitated EPS molecules were spun down and dried at 55°C oven overnight before determination of dry weight. Each experiment was repeated at least twice with triplicate. The data shown are the average of triplicate with standard deviation.

## Authors' contributions

JEW carried out all the HPLC and NMR analysis. JSC generated all the mutants. The study was conceived, designed, and coordinated by LHZ and YWH, who also drafted the manuscript and extracted all the DSF signals, and did the virulence factor production assay. All authors read and approved the final manuscript.

## Supplementary Material

Additional file 1 **MS analysis of DSF from *Xoo *strain KACC10331**. High-resolution electrospray ionization mass spectrometry was performed on a Finnigan/MAT MAT 95XL-T mass spectrometer. Click here for file

Additional file 2 **MS analysis of BDSF from *Xoo *strain KACC10331**. Click here for file

Additional file 3 **HPLC analysis of ethyl acetate extract from the supernatant of *rpfF *mutant cell culture**. The same volume of *rpfF *mutant supernatant was extracted for DSF-family signals using the same protocol as described in the Materials and Methods. (a) DSF, (b) BDSF, and (c) CDSF. Click here for file

Additional file 4 **Effects of different concentrations of DSF, BDSF and CDSF on EPS production and xylanase activity**. **(A) EPS production**. (B) The xylanase activity in the supernatant of cell culture. Click here for file
