# Supplementary figures and images for: Rice bacterial blight pathogen Xanthomonas oryzae pv. oryzae produces multiple DSF-family signals in regulation of virulence factor production
Source: BMC Microbiol. 2010 Jul 9;10:187. doi: 10.1186/1471-2180-10-187 (PMC2909994; doi:10.1186/1471-2180-10-187)

## Slide 1
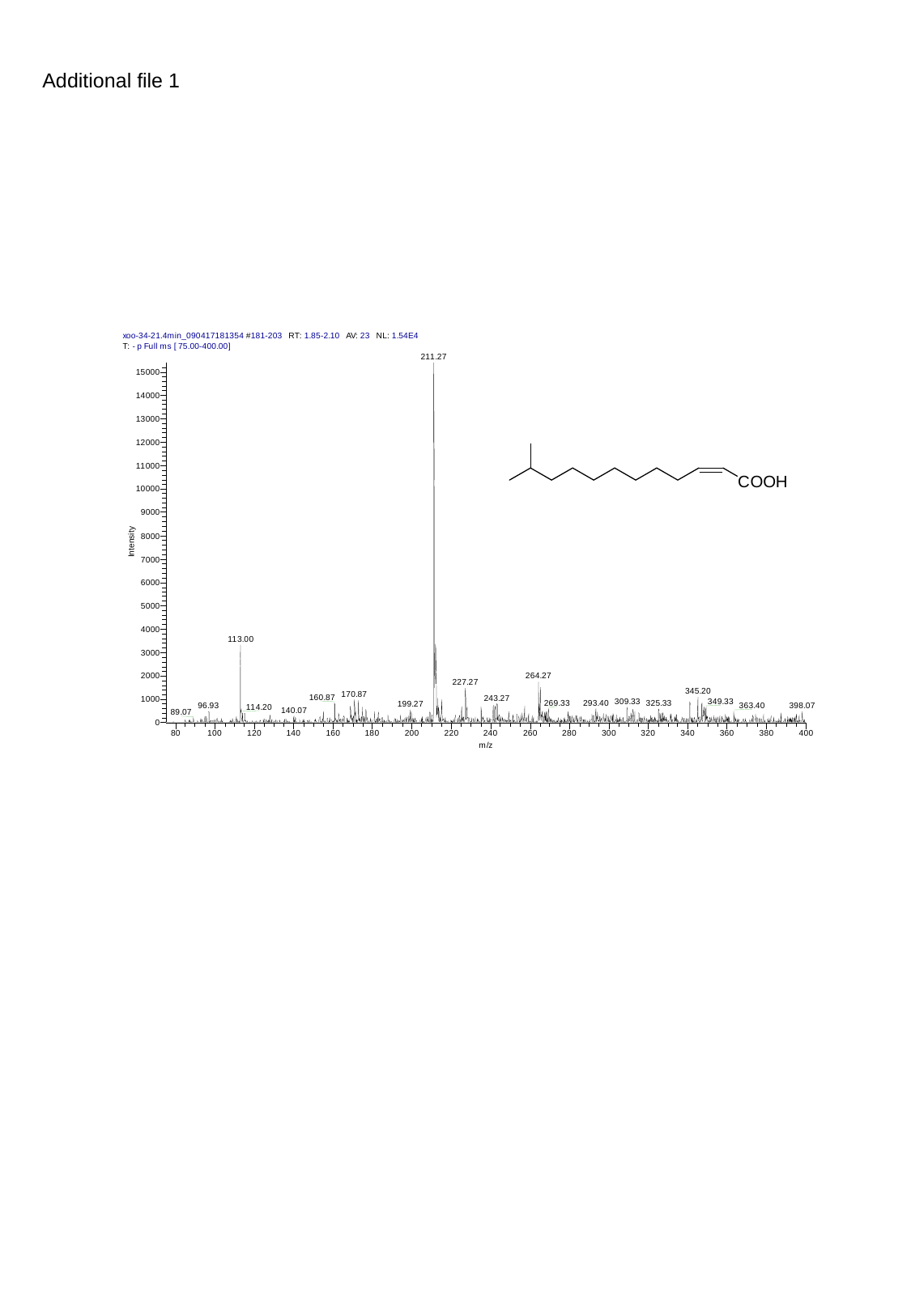

# Additional file 1

Supplement: Additional file 1 — MS analysis of DSF from Xoo strain KACC10331. High-resolution electrospray ionization mass spectrometry was performed on a Finnigan/MAT MAT 95XL-T mass spectrometer. [file 1471-2180-10-187-S1.PPT]

## Slide 1
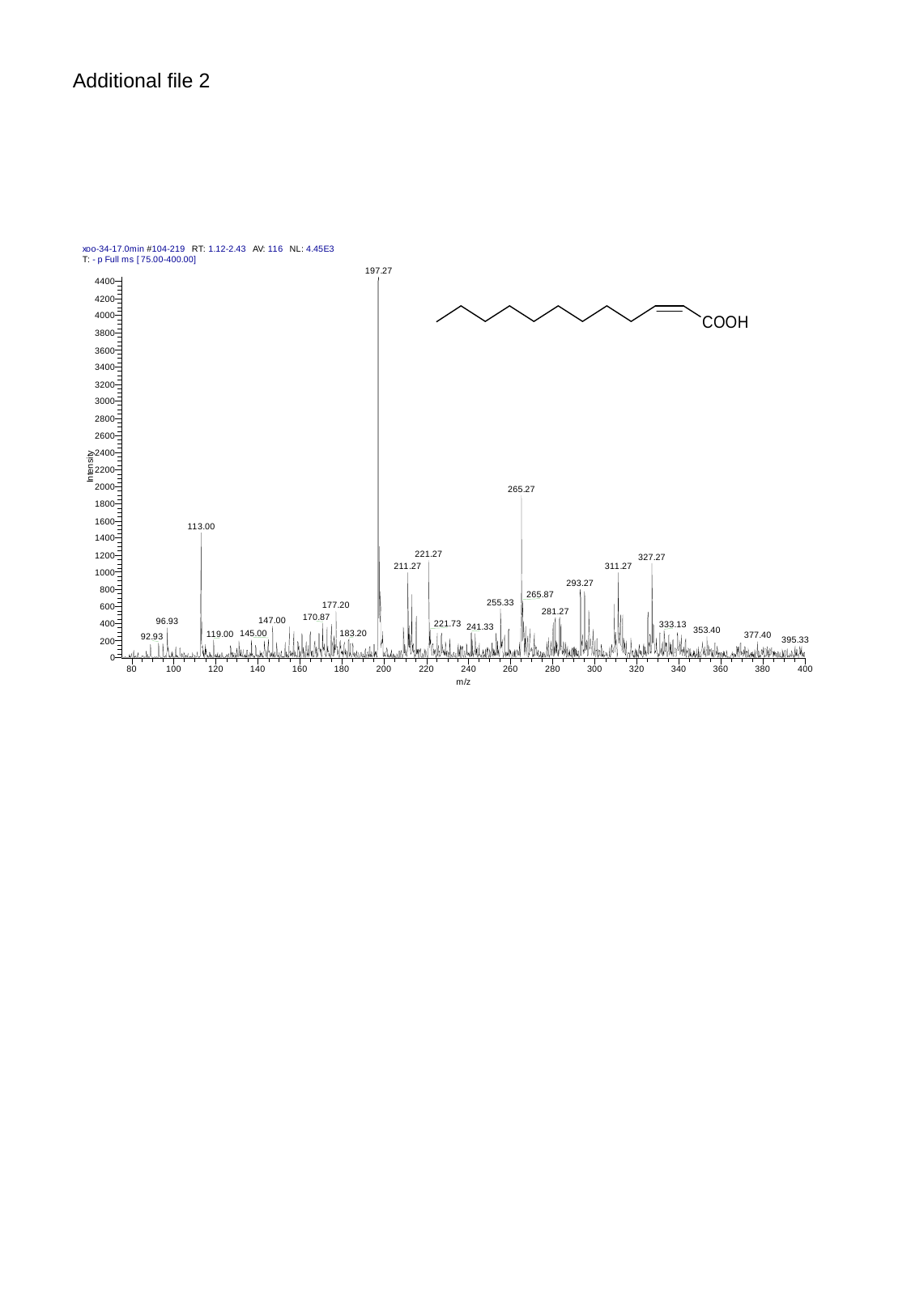

# Additional file 2

Supplement: Additional file 2 — MS analysis of BDSF from Xoo strain KACC10331. [file 1471-2180-10-187-S2.PPT]

## Slide 1
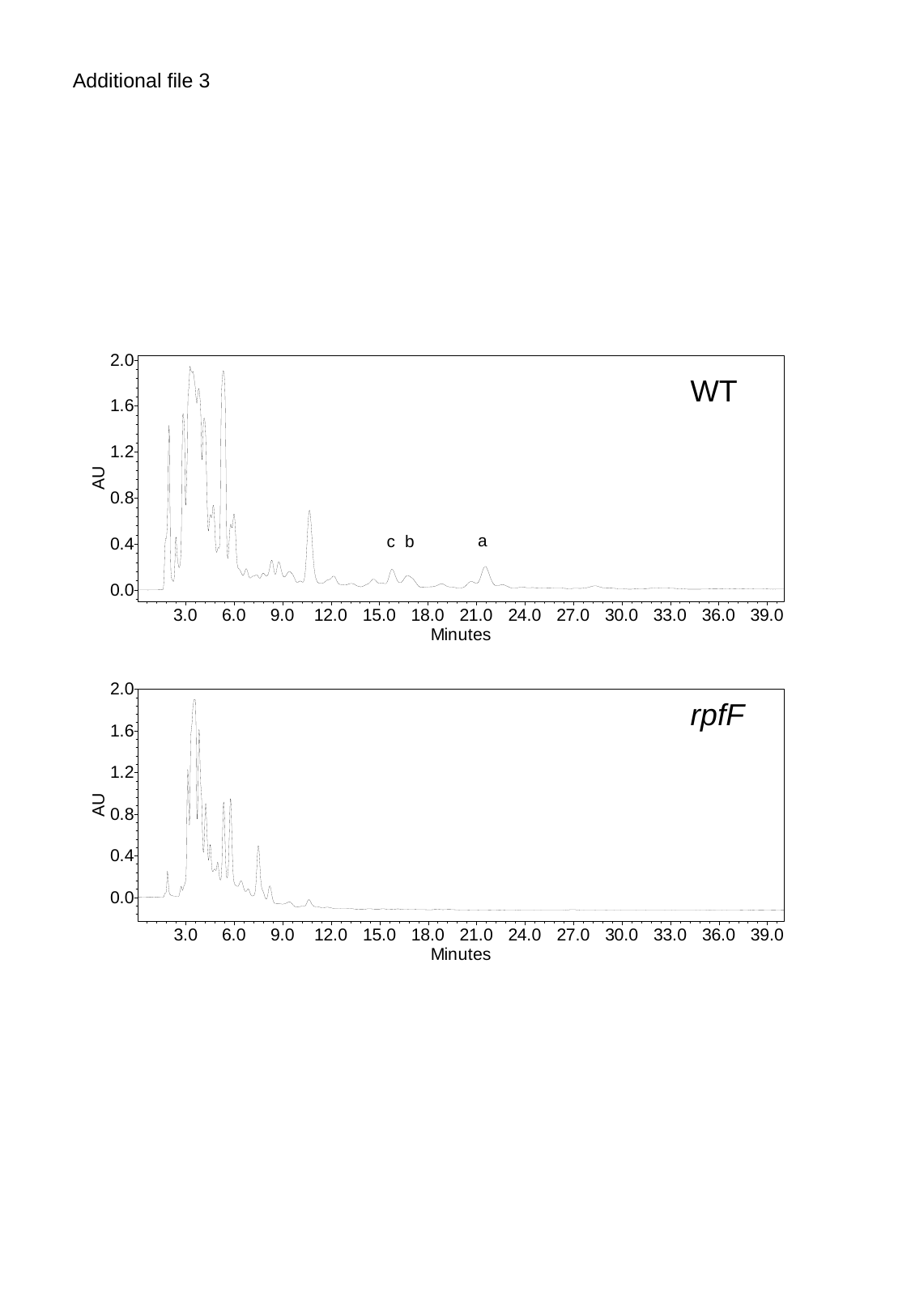

# Additional file 3
WT
a
c
b
rpfF

Supplement: Additional file 3 — HPLC analysis of ethyl acetate extract from the supernatant of rpfF mutant cell culture. The same volume of rpfF mutant supernatant was extracted for DSF-family signals using the same protocol as described in the Materials and Methods. (a) DSF, (b) BDSF, and (c) CDSF. [file 1471-2180-10-187-S3.PPT]

## Slide 1
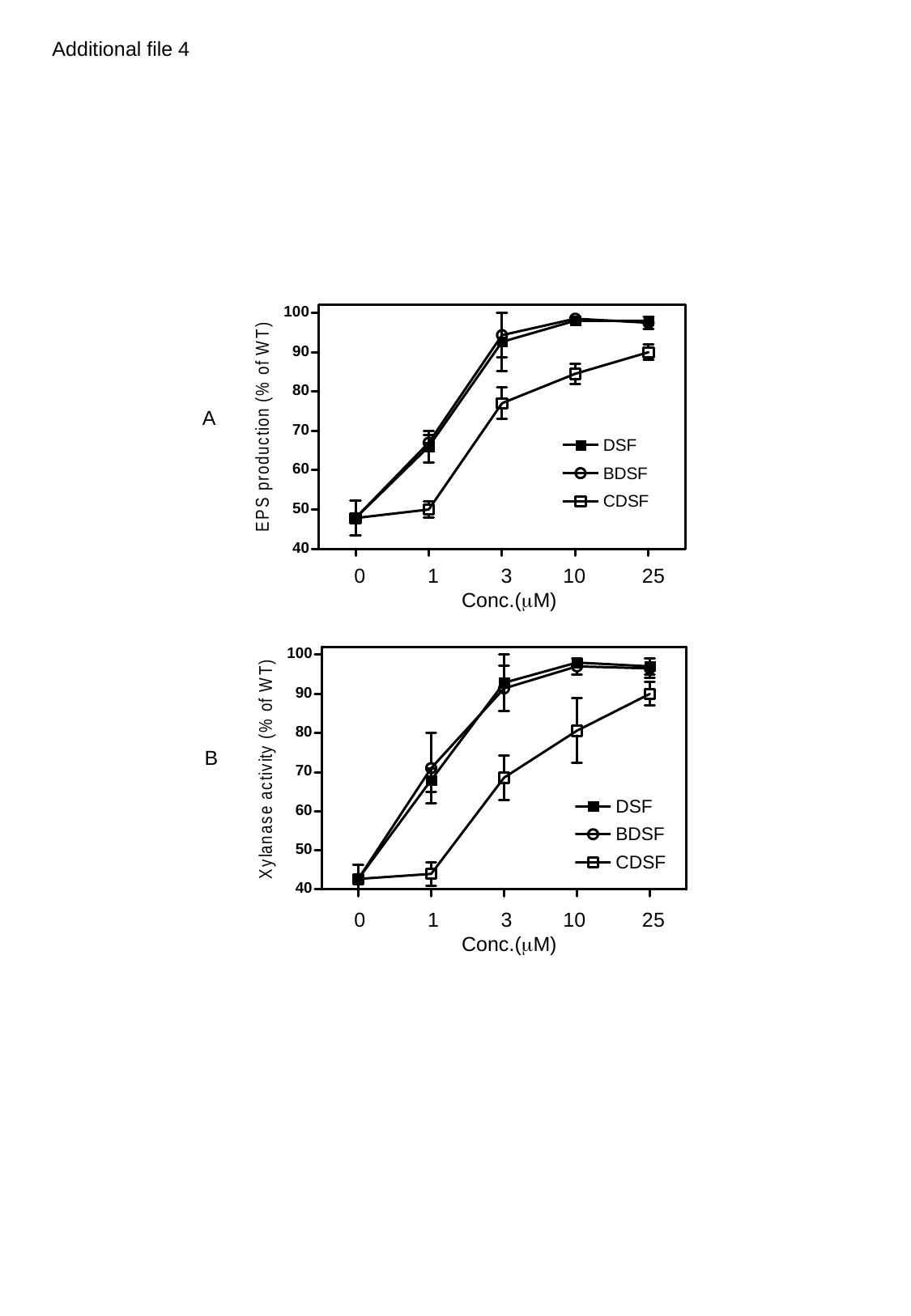

Additional file 4
A
 0 1 3 10 25
 Conc.(μM)
B
 0 1 3 10 25
 Conc.(μM)

Supplement: Additional file 4 — Effects of different concentrations of DSF, BDSF and CDSF on EPS production and xylanase activity. (A) EPS production. (B) The xylanase activity in the supernatant of cell culture. [file 1471-2180-10-187-S4.PPT]
